# Chilean physicians and the study of Peruvian wart: a critical review

**DOI:** 10.17843/rpmesp.2025.424.15401

**Published:** 2025-12-12

**Authors:** Oscar G. Pamo Reyna

**Affiliations:** 1 Universidad Peruana Cayetano Heredia, Lima, Peru. Universidad Peruana Cayetano Heredia Universidad Peruana Cayetano Heredia Lima Peru

**Keywords:** History of Medicine, Carrion’s Disease, Human Bartonellosis. Peruvian Verruga

## Abstract

Chilean physicians Nicolas Malo, Francisco Puelma Tupper, Luis Sanfurgo Reyes, and Vicente Izquierdo Sanfuentes published separate articles and theses on verruga disease between 1852 and 1886. However, in light of the facts, these publications made no contribution to the knowledge or national research on verruga disease because they were not accessed until many years after they were published.

## INTRODUCTION

Carrion’s disease or human bartonellosis is an infectious disease produced by *Bartonella bacilliformis* and transmitted by mosquitoes of the genus *Lutzomyia*, and it is endemic in certain valleys of Peru, Ecuador, and Colombia. This disease has three clinical phases: the anemizing fever, the intercalary period, and the verrucous phase, the latter being characterized by the appearance of angiomatous lesions of variable size on the skin of those affected.

Knowledge of the clinical course of human bartonellosis represented a challenge for Peruvian medical research, the trigger for which was the sacrifice of medical student Daniel Alcides Carrión in 1885, when he autoinoculated himself with blood from verrugas (warts) in his eagerness to know the prodrome of said disease, which cost him his life.

The objective of the present review was to determine the contribution of the publications made by Chilean physicians to the knowledge of Carrion’s disease.

### Nicolás Malo

In June 1895, Dr. Pablo Patrón Faustos addressed the secretary of the editorial staff of La Crónica Médica, Dr. Elías L. Congrains, sending him the text of the thesis of the Chilean physician Dr. Nicolás Malo, presented in Santiago, Chile in 1852, so that it might be reproduced, since said journal should constitute an archive of everything related to that little-studied disease that was Peruvian verruga. The text of said thesis was published on July 15, 1895, in issue 157 of said journal ^1^.

Dr. Pablo Patrón Faustos (1855-1910) was a physician from Lima interested in archaeology, linguistics, Peruvian history, and bibliographic studies, and it is unknown how he obtained said text.

To place ourselves in context, let us recall that the War of the Pacific declared by Chile against Peru, which was adverse to the latter, occurred between 1879 and 1883; that Carrión’s auto-experiment happened in 1885; and that around 1895 the country was in the process of national restoration.

In 1856, the bachelor of medicine Tomás Salazar published his doctoral thesis «Historia de las verrugas» (History of the verrugas)—considered the first scientific medical article of Peru—and in the historical background, he mentions only the chronicler Agustín de Zárate and makes no reference whatsoever to the publication of Dr. Malo ^2^.

During the discussions that arose with the death of Carrión in 1885, Dr. Malo’s thesis was not mentioned, so it is assumed that its reproduction in La Crónica Medica in 1895 made said information known for the first time in our environment.

Nicolás Malo graduated in 1846. He was a doctor of medicine and lieutenant of the Protomedicato in the department of Ancash in 1847 and, holding the same position, in the department of Puno in 1850. In 1852, he presented his thesis titled «Verruga peruana» (Peruvian Verruga) at the University of Chile ^3^.

In his thesis, Dr. Malo makes a quick differentiation between the verruga vulgaris, which we now know as papillomas, and the bleeding dermal lesions known as «verrugas» in Peru. He states that he observed them in the inhabitants of the northern and central highlands of Peru, as well as in people who traveled to the coast or moved to the capital of the Republic, and claims to have suffered from them for nine months. He considers that this disease affects people of all ages, especially males; it is distributed throughout the body; and the clinical course has four periods (hyposthenia; bone, articular, and muscular pain; appearance of the verrugas; and softening of the lesions and bleeding, disappearing without scars) which last several months and are not contagious. He mentions as predisposing factors fatigue, the elements, and the water of certain streams, the latter being a belief deeply rooted among the locals. For treatment, after several failed attempts, he recommends the use of energizers, diuretics, and diaphoretics. He also states that «The pathological anatomy remains to be done... », daring to extirpate a verruga to examine it:

*Upon examining the extirpated tumor, I found a true erectile tissue, composed of several cells and many highly interlaced thin vessels: the cells contained pure blood of an arterial character; the thinned and highly adherent skin could be separated from the tumor (sic)* (Malo, N. p. 207).

Dr. Malo’s thesis probably went unnoticed in Chile, where verruga disease is considered an exotic disease, since its distribution is limited to certain valleys of Peru, Ecuador, and Colombia, where the human reservoirs reside and the vector inhabits, identified only some decades later.

From the foregoing, it was only in 1895 that Peruvian physicians had full knowledge of Dr. Malo’s thesis, which had been published in Santiago, Chile, in 1852.

### Francisco Puelma Tupper (1850-1933)

**Figure 1:**
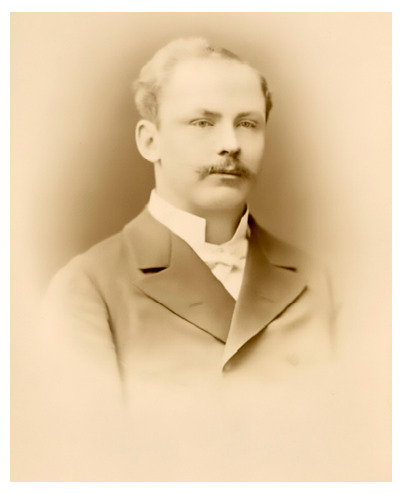
Francisco Puelma Tupper (1850-1933). National Museum of Medicine, Faculty of Medicine, University of Chile.

Source: https://n9.cl/1z0rl

Born in Santiago, he traveled to Europe in 1874 on a scholarship from the Chilean government to conduct studies in pathological anatomy. He was in Strasbourg, Vienna, and Berlin, and in the latter city, he worked with Dr. Rudolf Virchow. His thesis titled «La verruga peruana» allowed him to obtain the degree of doctor of medicine and surgery in Berlin, in May 1877 (Figure 1).

Puelma returned to Chile in 1879, at a time when the war against Peru had begun. He participated in field hospitals (*hospitales de sangre*), was appointed chief of Health of the Army and Navy, and designated professor of pathological anatomy at the School of Medicine in 1881. Shortly after, he also ventured into politics.

The thesis on Peruvian verruga was reproduced in *La Revista Médica de Chile* in 1878 ^4^^,^^5^. Said thesis is based on the observations of various authors, whom he cites frequently. Furthermore, Puelma mentions having participated in two autopsies of patients who had been in Peru and returned to Santiago with verrugas.

In the historical background, Puelma mentions the writings on verrugas by Tschudi, Hirsh, Smith, Dounon, Renaud, and Raimondi, among others. He cites the Peruvian physician Tomás Salazar, who approved and published his doctoral thesis titled «Historia de las verrugas» in 1858, although he does so erroneously, as he mentions him as M. Salazar and attributes the publication to the year 1860. Curiously, he does not mention his countryman, Dr. Nicolás Malo, who had presented his thesis on Peruvian verruga in Santiago in 1852, as mentioned above.

At the time Puelma was collecting material for his thesis in 1872, an intense debate existed in Peru regarding the nature of verruga disease and its relationship with the so-called anemizing fever or Oroya fever. However, he makes no reference whatsoever to the Peruvian experiences. He describes the coexistence of both diseases, fevers and verrugas, but does not dare to establish a conclusion:

*Furthermore, the verruga and the intermittent fever appear in the same region and at the same time, without it being possible to say that the former affection accompanies the latter, while it can be said that the latter accompanies the former, at least by being in the same region. Where the verruga is endemic, intermittent fever is always found; so that many unfortunate people have to endure both affections. (sic)* (Puelma Tupper, F. VII(11): 275).

It must be recognized that Puelma’s thesis is more orderly than Malo’s, but it reflects a great confusion typical of the era, when the nosography of diseases was still becoming known and the etiopathogenesis of verruga disease was unknown.

Regarding the pathological anatomy, Puelma mentions in his thesis:

*Only four autopsies of verruga cadavers have been performed. One of them was done by the aforementioned Salazar, and to avoid repetitions we transcribe it here. (sic)* (Puelma Tupper, F. VII(11): 300).

After reproducing Salazar’s autopsy, Puelma reports having participated in two autopsies performed in Santiago in 1872, when he was still a student. The findings showed hepatomegaly, splenomegaly, and the characteristic dermal lesions. However, other findings were also identified suggesting that said verrucous individuals did not die because of the verrugas, but due to complications. Currently, it is known that, in the verruga phase, the affected individual is in a state of immunodeficiency, making superinfections frequent.

Regarding histology, he claims to have performed a microscopic study of the verrugas and points out the following:

*...they consist of capillaries or larger vessels that present a cavernous type. Around these vessels, a cellular tissue is seen that recalls the embryonic, in intimate union with the wall of the vessel and without visible division of it. Often blood corpuscles and hematin crystals are seen between the meshes of the cellular tissue. Sometimes small hemorrhagic foci are found. All this is enclosed in a layer of skin and epidermis. The epidermis, however, is very thickened in the prominent part of the verruga;... (sic)* (Puelma Tupper, F. VII(12): 353).

Puelma did not agree with the denomination of «sarcomas» for the verrugas, as Dounon and Renaud did, and preferred to call them «blood warts», like Tschudi.

Summarizing, while still a student in 1872, Puelma compiled information on Peruvian verruga from reading previous articles, and participated in the autopsies of two Chilean citizens who had returned to their country with verrugas:

*We can add that in Chile no case of infection of verruga is known despite the many countrymen who return from Peru attacked by it. (sic)* (Puelma Tupper, F. VII(11): 276).

In this way, Puelma had sufficient material to present it as a doctoral thesis while in Berlin in 1877.

Since Puelma’s thesis was published in Santiago, in volume VII, issues 11 and 12 of *La Revista Médica de Chile*, corresponding to the years 1878-1879, it is probable that this information did not reach Peru due to the war in progress and the blockade of the port of Callao by the Chilean squadron. Indeed, between April 5 and August 8, 1879, naval incursions by both sides occurred, culminating in the loss of the main Peruvian ship at Punta Angamos. Subsequently, Chile established the naval blockade of Callao, which extended from April 10, 1880, to January 17, 1881, preventing the entry and exit of ships in said port.

### Luis Sanfurgo Reyes

Born in 1859, he arrived in Lima with the occupation of the city by Chilean forces, as part of the military health corps, in the second half of January 1881, and remained until the end of 1883, when the invading troops withdrew.

Sanfurgo observed that many Chilean soldiers developed Peruvian verruga, which motivated him to dedicate his thesis to this topic. This was foreseeable, given that Chilean troops made incursions into the verruga-endemic valleys.

His thesis, titled «La verruga peruana i su tratamiento» (Peruvian verruga and its treatment), presented as a memoir, was read in Santiago on February 20, 1885, which allowed him to obtain the licentiate in medicine and pharmacy ^6^.

Judging by the content of his work, Sanfurgo knew the articles previously published on Peruvian verruga, and does not skimp on mentioning his countrymen Nicolás Malo and Francisco Puelma Tupper.

Sanfurgo’s monographic thesis constitutes a good review of what was known about Peruvian verruga up to then, and is drafted in an orderly manner, comparable even with the work of Tomás Salazar published in 1858, whom he also mentions.

From his memoir, it is inferred that, in Lima, Sanfurgo provided services at the Santa Sofía Hospital, then converted into a military hospital exclusively for the wounded and sick of the Chilean occupation army ^7^. This hospital was built between 1872 and 1876, financed by the French businessman Auguste Dreyfuss, in memory of his wife Sofía Bergmann, who died in 1871. It was located on the premises of the current José Pardo Public Technological Higher Education Institute.

Sanfurgo conducted microscopic and experimental studies of the water from the Quebrada de Verrugas, in an attempt to find a possible causal agent:

*The microscopic analysis has not yet given positive results and only remains of vegetables and some infusoria have called our attention. Hypodermic injections practiced with those waters in guinea pigs and rabbits have not given results either. (sic)* (Sanfurgo Reyes, L. p. 521).

Sanfurgo was aware of the discussions on the etiological unity of the disease, that is, that the anemizing fever and verruga disease obey the same agent, but he does not agree with that position:

*“Some have believed to find certain similarity of development and of action between the agent of this disease and that which produces the intermittents; but, if this similarity may be true for the former, it is not, on the other hand, for the latter, since the anatomo-pathological lesions, the symptoms, the course and treatment and even the physiognomy itself of both diseases are entirely different; and if by the change of place or climate the two agents are destroyed for not being able to live outside the conditions in which they were born, they do not for this reason cease to be distinct pathological individualities that modify the organism in an entirely diverse manner.” (sic)* (Sanfurgo Reyes, L. p. 521).

Sanfurgo describes the macroscopic examination of a cut verruga, but did not perform microscopic studies, which is deduced from:

*“From the microscopic examination practiced by Messrs. Dounon and Renaud, and lately by Professor Izquierdo, it results that the superficial dermal tumors originate in the papillae themselves and raise and thin the epidermis that covers them considerably. They are formed by cavernous tissue of circular areolas, full of blood during life and empty afterwards, and whose trabeculae are very rich in cells.” (sic)* (Sanfurgo Reyes, L. p. 518).

At this point, it is worth highlighting that Sanfurgo refers to Dr. Vicente Izquierdo, whom he likely contacted at the end of the war, in 1884, and from whom he obtained the information he cites in his thesis. It was Sanfurgo himself who provided the verruga samples for Izquierdo to examine, which allowed the latter to identify a supposed “schizomycete” as a possible causal agent:

*It was, then, very possible that the verruga would also be found in analogous conditions of genesis as the paludal intermittents. Influenced by these ideas, we collected with the greatest care a quantity of tumors of diverse forms, taken either during the life or after the death of the patients and which we preserved in alcohol to entrust them to Professor Don Vicente Izquierdo, who has made a prolix study of them and found, finally, the cause of this special disease in the presence of a schizomycete.*

*Of the class of micrococci, this schizomycete often affects the form of a bacillus by grouping in rows and in variable number, rows that are almost never rectilinear but in zig-zag or in the form of an S. Its size is about half that of gonococci and they are round or slightly elliptical when they are in a row. They are found in the structural tissue of the tumors between the cells, forming small colonies and never inside them. It is also found inside the capillaries and small arterial and venous vessels of the apparently healthy skin and of that covering the nodes, in the capillaries of these and in those of the subcutaneous cellular tissue, finding many of those vessels entirely thrombosed by compact masses of micrococci that dilate them irregularly to the point of resembling lymphatic vessels and observing very often around them the beginnings of a cellular proliferation, origin of the tumors. It is these micro-beings that, as in tuberculosis, leprosy, etc., producing a cellular irritation in the connective tissue of the skin and mucosae, originate the tumors of this disease and the alteration of the blood, since they circulate with it. (sic)* (Sanfurgo Reyes, L. p. 522).

In that era, bacteria were called «schizomycetes», from the Greek: «fungi that divide», as it was thought they were a type of fungus due to their reproduction by fission or cell division. Was what Izquierdo saw any microbe, according to Sanfurgo? Were they bacilli in rows, elliptical and almost elliptical, in zigzag or S rows?.

Currently, it is known that bartonellas are bacilli and coccobacilli, but they do not group in rows. A gonococcus measures on average 0.8 microns, while the length of a *Bartonella* is 2 to 3 microns. These bacteria are intracellular and lodge in the endothelial cells of the verrucoma, without forming endovascular thrombi. Therefore, it is unlikely that Izquierdo observed the true causal agent of Peruvian verruga.

Given that Sanfurgo presented his thesis on February 20, 1885, it is reasonable to suppose he received the information from Izquierdo during 1884. Curiously, although Sanfurgo was in Lima between 1881 and 1883 attending to the wounded and sick of the Chilean army, and interested in the cases of verruga among the soldiers, local physicians do not mention him until 1898.

Summarizing, Sanfurgo performed a detailed description of Peruvian verruga in his thesis, incorporated the information provided by Izquierdo, and hinted at a possible microbial cause of the disease. However, Sanfurgo’s thesis went unnoticed in Peru, where he was not cited until many years after 1885, the year in which Carrión’s auto-experiment occurred.

### Vicente Izquierdo Sanfuentes (1850-1926)

**Figure 2:**
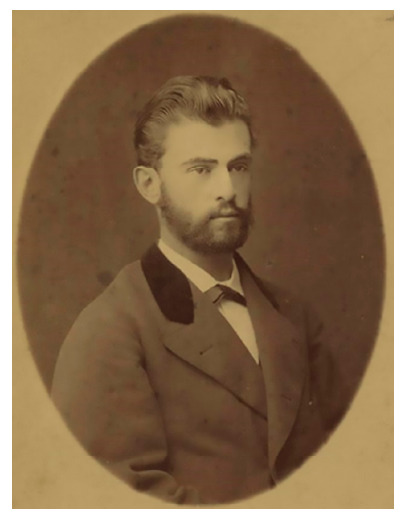
Vicente Izquierdo Sanfuente (1850-1926). National Museum of Medicine, Faculty of Medicine, University of Chile.

Source: https://n9.cl/5rzjz

He was academically trained in Germany, where he completed his medical and scientific education. He graduated as a physician in 1879 and returned to Chile in the midst of the War of the Pacific (1879-1883). There he revalidated his medical degree and was appointed professor of histology at the University of Chile in 1881. That same year he traveled again to Germany and returned in 1883 (Figure 2).

In 1885, Izquierdo published an article in the prestigious German journal *Virchows Archiv*, on his research in which he highlighted the finding of a presumed causal agent of Peruvian verruga («Spaltpilze bei der “Verruga peruana”»), a disease that had awakened his interest for having affected Chilean soldiers during the war ^8^.

In his article, Izquierdo mentions how he obtained the samples:

*It is known that the infectious disease, endemic in Peru and known as “verruga peruana”, does not occur in Chile. The few cases treated in Santiago hospitals come from Peru and most are cured. Therefore, it is very difficult to obtain fresh material for histological examination. Recently I received from Lima some pieces of skin from the knee area and the foreskin, which showed several nodules similar to the verruga. The material was very well preserved in alcohol and ten pieces were taken after the death of an individual who had succumbed to the disease. (sic, trans.)* (Sanfurgo Reyes, L. p. 522).

Izquierdo highlighted, as was already known, the vascular nature of the verrucous lesion:

*Thus, the verruga nodules resemble sarcomas in their structure, without this being a reason to consider them as such, as Dounon and Renaud do, since verrugas, as we shall see shortly, arise as a result of the irritation of the connective tissue by a fission fungus, which is probably peculiar to this disease. In this sense, they are similar to the nodules of leprosy and tuberculosis. All nodules are very rich in blood vessels; a large number of capillaries run through the tissue in all directions and a good part of them are obstructed by a large number of bacteria. In some nodules the vessels, especially in their middle part, are very thick and are completely full of red blood cells; in other places, a true cavernous tissue develops, in which the blood fills large cavities, separated from each other by anastomosed fibrous walls; the similarity with a true cavernous angioma is complete (sic, trans.)* (Izquierdo V. p. 413).

As was to be expected for the era, Izquierdo sought a microbial agent in Peruvian verruga:

*It is natural that I have sought fission fungi in the pathological product of an infectious disease, using the new methods that science possesses to detect the presence of bacteria in the tissues. My investigations have led me to the discovery of a fission fungus of the Bacillus form, which, due to its intimate connection with the tissue of the nodules, I thought was the cause of the products of the disease, and which was probably the cause of the disease itself. This Bacillus is found both in the nodules themselves and in the apparently healthy skin that surrounds them. It stains with various aniline colors, with fuchsia, methyl violet, methylene blue, etc., everything stains nothing or is as pretty as gentian violet. Now we apply this color to all. The preparations of each of the methods, by means of which Koch’s bacillus is stained, develop completely in acids, which leave the coloration of the tubercle bacillus unaltered. In a later work I will give more details on the technique.*

*The Bacillus found can reach up to 20 μ in length, but this is rare; most measure 8-12 μ, and considerably smaller ones can be found. They are somewhat thicker than Koch’s tubercle bacilli. The great diversity of lengths is probably related to the different phases of development or spore formation; the longest are always found in the tissue of the nodules, as are the smallest, while those of medium size are usually found in healthy skin or in blood vessels close to the ulcers. (sic, trans.)* (Izquierdo V. p. 414).

Izquierdo described grouped bacilli and cocci, extracellular and thrombosing the capillaries, exactly as Sanfurgo described (see above):

*In the tissue of almost all the nodules there is always a large quantity of the described fission fungi, of all sizes, in groups between the cells, very rarely individual, and I have never seen them inside the cells. These groups are formed by more or fewer individuals, which often are very long and are closely intertwined. These long bacilliform formations are almost never straight, but are always bent at more or less acute angles, forming a zig-zag or an S. In some places the tissue is diffusely permeated by individual cocci or two or more connected cocci, as if the microbes had completely dissolved into the granules they form. In these places, diffusely penetrated by cocci or small chains of cocci, the tissue cells are destroyed and in their place dense masses of cocci are found. In the center or the periphery of most of the examined ganglia I found a large number of capillary vessels and probably also small veins that were completely blocked by compact microbial masses. There are true networks of these vessels, which seem to be injected with masses of bacteria and, therefore, completely thrombosed.” (sic, trans.)* (Izquierdo V. p. 415).

From this description, it is deduced that Sanfurgo received this information from Izquierdo in 1884 and copied it ad literam.

Izquierdo’s conclusions were the following:

*(1) The tumors that form on the skin of patients suffering from verrugas are true neoplasms of the connective tissue, which always develop in the skin or in the subcutaneous cellular tissue. They are never warts in the anatomical sense. 2. Their structure is very similar to that of sarcomas; in many of them parts formed solely by cavernous tissue are found. (3) In these neoplasms a certain type of fission fungus is found in large quantities, which is either found among the anatomical elements, such as cells or fibers, or is contained in the blood vessels, which sometimes obstruct them completely. (4) The same fission fungus is found in the blood vessels (capillaries and veins) of healthy skin and subcutaneous cellular tissue. 5. It is also found in the tissue and vessels of the skin covering the nodules. 6) Thus, in the blood of patients with verrugas circulates a schizomycete which, by stimulating the connective tissue, causes the formation of new cells in specific and determined places, thus causing the development of the nodules, which are erroneously called “verrugas”. 7 It cannot be assumed that this fission fungus only developed after death; the large number of vessels thrombosed by the microbes and their presence in the vessels of healthy skin indicate otherwise. (sic, trans.)* (Izquierdo V. p. 418).

What was described by Izquierdo and repeated by Sanfurgo contrasts with what became known later about bartonellas: they are intracellular bacillus or coccobacillus type bacteria, located in the red blood cells during the anemizing phase and in the endothelial cells of the verrucous formations or verrucomas, and do not form groupings that thrombose blood vessels.

It was an era in which there was a quest to identify the causal agents of known diseases. Thus, we have that the following discoveries had already been made: chicken cholera (1868), leprosy (1873), intestinal amebiasis (1875), anthrax (1876), gonorrhea (1879), typhoid fever (1880), malaria (1880), tuberculosis (1883), cholera (1883), diphtheria (1884), and tetanus (1885). Therefore, an attempt had to be made to discover a causal agent for Peruvian verruga.

In Peru, microscopic pathological anatomy studies only began to develop systematically towards the end of the 19th century. In 1899, Manuel Tamayo Möller presented his bachelor’s thesis titled «Histología patológica de la verruga nodular» (Pathological histology of nodular verruga) ^9^. That same year, Oswaldo Hercelles Monterola presented his work «Histología patológica del noduloma verrucosos» (Pathological histology of the verrucous noduloma), where he defends the vascular theory as the origin of the verruga and mentions the existence of «germs» causing Oroya fever and the verrugas, although they had not yet been identified ^10^. Shortly after, in 1901, Hercelles installed his new laboratory of chemistry, hematology, pathological anatomy, and bacteriology at the Dos de Mayo Hospital in Lima.

On the other hand, in bacteriological studies leading to identifying the causal agent of verruga disease, Alberto Barton published his findings of “endoglobular bodies” in the red blood cells of the anemizing phase of Carrion’s disease in 1905, which were confirmed in 1909 ^11^.

Izquierdo’s effort to try to find an etiological agent of Peruvian verruga in an era when different microorganisms were being discovered as causes of diseases must be recognized. However, his findings were not confirmed by later studies. Both technical limitations (such as the preservation and fixation of samples, appropriate stains, the degree of resolution of microscopes, etc.) and the limitations of the knowledge of the era, in particular the classification and typing of microorganisms, worked against him.

Carrión immolated himself on October 5, 1885, by inoculating himself with blood from a Peruvian verruga lesion, in his eagerness to know the prodromal symptoms of the disease. The evolution of his suffering was recorded in detail by his companions, who published the account one year after his death, in October 1886, in the book narrating what happened ^12^.

In the biographical note drafted by his companions, it is recorded that Carrión, supposedly, said:

*...well, it upsets me to see that individuals like the Chilean physician Izquierdo, who barely had a few tumors to see, launch into giving opinions, into writing about a disease that no one better should make known, for apart from the works of Drs. Salazar and Velez I have not heard speak of any other national;... (sic)* (Medina C. *et al.* p. 11).

Did Carrión really know of Izquierdo’s inquiries in 1885? Or was it something his companions learned in the following months and put said phrase on Carrión’s lips? It is important to define this, since some authors have supposed that Izquierdo’s study was a spur for Carrión to proceed with his auto-experiment. There are even authors who place nationalism at the forefront to explain Carrión’s determination to inoculate himself.

However, this hypothesis proves unconvincing. Carrión had gathered nine medical records of verruga patients during the previous three years, published an article on the urine of verruga patients, wished to know the prodrome of the disease, and inoculated himself with the sole objective of collecting information for his thesis, since he was in the final year of medical school.

The article in question by Izquierdo was published in Berlin, in the German language, in volume 99 of the journal corresponding to March 1885. Carrión immolated himself at the beginning of October of that same year. At that time, the distribution of foreign publications was carried out by sea and land distribution was slow. Furthermore, journals only reached subscribers, which implied an investment that many were not in a position to make.

It is very probable that said article circulated, in the best of cases, orally between 1885 and 1886. It should be remembered that, in Peru, since the mid-19th century, the influence of French medicine predominated, while Izquierdo’s publication was made in a German journal. Therefore, it proves unlikely that Carrión came to know Izquierdo’s publication.

It is probable that his companions embellished Carrión’s biography by including said detail in the 1886 publication. On the other hand, as already mentioned, Sanfurgo’s thesis of 1885, in which reference is made to Izquierdo’s work, became known much later, through the historical background presented by Dr. Ernesto Odriozola in 1898 ^13^.

Regarding the disease of Peruvian verruga, towards the end of the 19th century and during the first decades of the 20th century, national and foreign studies gradually defined the nosography, histopathology, causal bacterial agent, and vector. There were various observations and publications abroad made by travelers and physicians that were not known in Peru until many years later. Therefore, they did not contribute directly to the development of national investigations on Carrion’s disease, with its anemizing and verrucous phases.

References on Peruvian verruga began to be mentioned after 1885, the year of Carrión’s auto-experiment. It was thus that the references of the chroniclers of the Conquest, the verruga of the liberating troops, and the experiences of foreign travelers began to be studied. One of the first reviews was that of Dr. Ernesto Odriozola.

In 1898, Dr. Odriozola published his book «La maladie de Carrion» (Carrion’s Disease), probably the most complete study of the era for said disease ^13^. In the historical background, he refers to Malo in the following manner:

*We consider that this thesis, despite its numerous errors and omissions, is of great merit. We must not forget that it is the first attempt on the subject and, however imperfect it may be, it demonstrates a spirit of clinical investigation; and the contradictions found therein are only those of an experience still rudimentary for the era. (sic, trans.)* (Medina C. *et al.* p. 11).

Regarding Puelma Tupper, for his 1877 thesis, after making several observations, he concludes the following:

*We do not wish to criticize other errors, which were very understandable given the era in which this work was published. But we want to make it clear that this thesis seems worthy of being held in high esteem and the author demonstrates a very serious clinical judgment. (sic, trans.)* (Odriozola E. p. 31-32).

With respect to Sanfurgo, after raising objections to various aspects of his work, and referring to the treatment of verrugas, Odriozola writes:

*In the chapter on treatment, he states, or at least lets us guess, that he was the first to use arsenic and iodine, and adds that the latter gave him excellent results both internally and externally. We want to stick to the chronology of the facts on this point and demonstrate that forty years ago our old practitioners, Ríos Macedo, Odriozola, Villar, Olaechea, Espinal, etc., used arsenic and iodine without the slightest result and in hundreds of cases. (sic, trans.)* (Odriozola E. p. 32-33).

And, regarding Izquierdo’s publication, Odriozola concludes:

*Furthermore, the bacteriological examination was not carried out with the required scientific rigor. His observations focused on verrugas extirpated without precaution and which had remained in alcohol for a long time. From this, it clearly follows that we are authorized to formulate great reservations about his conclusions. (sic, trans.)* (Odriozola E. p. 34-35).

After an extensive historical, geographical, epidemiological, and clinical review of verruga disease, Odriozola, who was an eminently clinical physician, reserved the chapter on microscopic pathological anatomy for his professor, Dr. Maurice Letulle, of the Faculty of Medicine of the University of Paris.

Professor Letulle made a detailed description of the microscopic anatomy and regarding the supposed agent found by Izquierdo was very cautious in concluding:

*Awaiting the results of the experiments and cultures carried out by Professor Odriozola, we may ask ourselves, together with Izquierdo, if the bacillus represented in plate VIII, fig. 2, is not the pathogenic element of the verruga.*

*In the future, it will be considered as a saprophytic bacillus comparable to the numerous parasites of human skin, or as the primordial element of the endemic verrucous disease, so notably described in the book we have just read. (sic, trans.)* (Odriozola E. p. 201-210).

In conclusion, it is important to rescue the interest of the Chilean physicians Nicolás Malo, Francisco Puelma Tupper, Luis Sanfurgo Reyes, and Vicente Izquierdo Sanfuentes in making known and publishing on verruga disease, despite the limitations inherent to the era (Table 1). For them, it was an unknown and exotic disease, and they did not publish on the subject afterwards. Consequently, their publications were isolated works that were discovered subsequently by national authors, who mentioned them in the bibliography of the historical background of verruga disease, as they became known. Therefore, none of said publications constituted a substantive contribution nor had a direct impact on Peruvian medical investigations on the anemizing fever and the verrucous phase, which today are recognized as two manifestations of the same disease.

**Table 1:** Chilean physicians who published on Peruvian Verruga (Wart) in the 19th century.

	Year of publication	Type of publication	Location	Title	Was in Peru
Nicolás Malo	1852	Bachelor’s thesis	Santiago de Chile	Verruga peruana	Yes
Francisco Puelma Tupper	1879	Doctoral thesis in medicine and pharmacy	Berlin	La verruga peruana	No
Luis Sanfurgo Reyes	1885	Dissertation to obtain the degree of Licentiate in medicine and pharmacy	Santiago de Chile	La verruga peruana i su tratamiento	Yes
Vicente Izquierdo Sanfuentes	1885	Article	Berlin	Esquizomicetos en la verruga peruana	No

## References

[1] Malo N (1895). Verruga peruana.

[2] Salazar T (1858). Historia de las verrugas. Gaceta Médica de Lima.

[3] Valdizán H (1959). Nicolás Malo. Diccionario de Medicina Peruana.

[4] Puelma Tupper F (1878). La verruga peruana. Revista Médica de Chile.

[5] Puelma Tupper F (1879). La verruga peruana. Revista Médica de Chile.

[6] Sanfurgo Reyes L (1885). La verruga y su tratamiento. Anales de la Universidad de Chile.

[7] Deza Bringas L (2004). Santa Sofía el hospital que nunca fue. Rev Neuropsiquiatr.

[8] Izquierdo V (1885). Spaltpilze bei der "Verruga peruana". Archiv f pathol Anat.

[9] Tamayo MC (1899). Histología patológica de la verruga nodular.

[10] Hercelles O (1899). Histología patológica del noduloma verrucoso. La Crónica Médica.

[11] Barton A (1909). Descripción de los elementos endoglobulares hallados en los enfermos de fiebre verrucosa. La Crónica Médica.

[12] Medina C, Mestanza E, Arce J, Alcedán M, Miranda R, Montero M (1886). La verruga peruana y Daniel A. Carrión.

[13] Ernesto Odriozola (1898). La maladie de Carrion ou la verruga péruvianne.

