# Supplementary material for: Chilean physicians and the study of Peruvian wart: a critical review
Source: Rev Peru Med Exp Salud Publica. 2025 Dec 12;42(4):447–54. doi: 10.17843/rpmesp.2025.424.15401 (PMC12879972; doi:10.17843/rpmesp.2025.424.15401)
Supplement: Translation

# Translation: Los médicos chilenos y el estudio de la verruga peruana: una revisión crítica

**Authors:** Oscar G. Pamo Reyna

**Affiliations:** 1 Universidad Peruana Cayetano Heredia, Lima, Perú.

**Keywords:** Historia de la Medicina, Enfermedad de Carrión, Bartonelosis humana, Verruga Peruana

## Abstract

Los médicos chilenos Nicolás Malo, Francisco Puelma Tupper, Luis Sanfurgo Reyes y Vicente Izquierdo Sanfuentes publicaron sendos artículos y tesis sobre la enfermedad de verrugas, entre 1852 y 1886. Sin embargo, a la luz de los hechos, dichas publicaciones no significaron aporte alguno al conocimiento ni a las investigaciones nacionales sobre la enfermedad de verrugas debido a que no se tuvo acceso a ellas sino hasta muchos años más tarde de ser publicadas.

## INTRODUCCIÓN

La enfermedad de Carrrión o bartonelosis humana es una enfermad infecciosa producida por la *Bartonella bacilliformis* y transmitida por mosquitos del género *Lutzomyia* y es endémica en ciertos valles de Perú, Ecuador y Colombia. Esta enfermedad tiene tres fases clínicas: la fiebre anemizante, el periodo intercalar y la fase verrucosa, caracterizada esta última por la aparición de lesiones angiomatosas de tamaño variable en la piel de los afectados.

El conocimiento del curso clínico de la bartonelosis humana significó un reto para la investigación médica peruana, cuyo detonante fue el sacrificio del estudiante de medicina Daniel Alcides Carrión, en 1885, al autoinocularse sangre proveniente de verrugas en su afán por conocer el pródromo de dicha enfermedad, lo que le costó la vida.

El objetivo de la presente revisión fue determinar cuál fue el aporte o contribución de las publicaciones realizadas por los médicos chilenos al conocimiento de la enfermedad de Carrión.

### Nicolás Malo

En junio de 1895, el doctor Pablo Patrón Faustos se dirigió al secretario de la redacción de La Crónica Médica, Dr. Elías L. Congrains, remitiéndole el texto de la tesis del médico chileno Dr. Nicolás Malo, presentada en Santiago de Chile en 1852, para que se reprodujera toda vez que dicha revista debería constituirse en un archivo de todo lo relacionado con aquella enfermedad tan poco estudiada como era la verruga peruana. El texto de dicha tesis fue publicado el 15 de julio de 1895 en el número 157 de dicha revista ^1^.

El Dr. Pablo Patrón Faustos (1855-1910) fue un médico limeño, interesado en la arqueología, lingüística, historia peruana y estudios bibliográficos y se desconoce la manera de cómo consiguió dicho texto.

Para ubicarnos en el contexto, recordemos que la Guerra del Pacífico declarada por Chile al Perú, y que le fue adversa a este último, ocurrió entre 1879 y 1883, que la autoexperiencia de Carrión sucedió en 1885 y que hacia 1895 el país se hallaba en el proceso de restauración nacional.

En 1856, el bachiller de medicina Tomás Salazar publicó su tesis de doctorado «Historia de las verrugas» -considerado el primer artículo científico médico del Perú- y en los antecedentes históricos menciona solo al cronista Agustín de Zárate y no hace referencia alguna a la publicación del doctor Malo ^2^.

Durante las discusiones surgidas con la muerte de Carrión, en 1885, no se mencionó la tesis del doctor Malo por lo que se asume que su reproducción en La Crónica Medica, en 1895, dio a conocer dicha información por primera vez en nuestro medio.

Nicolas Malo se graduó en 1846. Fue doctor en medicina y teniente del Protomedicato en el departamento de Ancash en 1847 y, con el mismo cargo, en el departamento de Puno en 1850. en 1852 presentó su tesis titulada «Verruga peruana» en la Universidad de Chile ^3^.

En su tesis, el doctor Malo hace una rápida diferenciación de la verruga vulgar, que ahora conocemos como papilomas, de las lesiones dérmicas sangrantes conocidas como «verrugas» en el Perú. Manifiesta que las observó en los pobladores de la sierra norte y central del Perú, así como en personas que viajaron a la costa o se desplazaron a la capital de la República, y afirma haberlas padecido durante nueve meses. Considera a esta enfermedad afecta a personas de todas las edades, especialmente a los varones, se distribuye en todo el cuerpo y la marcha clínica tiene cuatro períodos (hipoestenia; algias óseas, articulares y musculares; aparición de las verrugas; y, ablandamiento de las lesiones y sangrado, desapareciendo sin cicatrices) que duran varios meses y que no son contagiosas. Menciona como factores predisponentes la fatiga, la intemperie y el agua de ciertos arroyos, creencia esta última muy arraigada entre los lugareños. Para el tratamiento, luego de varios intentos fallidos, recomienda el uso de energizantes, diuréticos y diaforéticos. También afirma que «La anatomía patológica está por hacerse;...»*,* atreviéndose a extirpar una verruga para examinarla:

*Examinado el tumor estirpado, encontré un tejido eréctil, verdadero, compuesto de varias celdillas y muchos vasos delgados sumamente entrelasados: las celdillas contenían sangre pura y de carácter arterial; el cutis adelgazado y sumamente adherido que podía separarse del tumor (sic)* (Malo, N. pág. 207).

La tesis del doctor Malo probablemente paso desapercibida en Chile, donde la enfermedad de verrugas es considerada una enfermedad exótica, ya que su distribución se limita a ciertos valles de Perú, Ecuador y Colombia, donde radican los reservorios humanos y habita el vector, identificado recién algunas décadas después.

De lo expuesto, recién en 1895 los médicos peruanos tuvieron un cabal conocimiento de la tesis del doctor Malo, que había sido publicada en Santiago de Chile en 1852.

### Francisco Puelma Tupper (1850-1933)

Nacido en Santiago, viajó becado por el gobierno chileno a Europa en 1874 para realizar estudios de anatomía patológica. Estuvo en Estrasburgo, Viena y Berlín, y en esta última ciudad trabajó con el doctor Rudolf Virchow. Su tesis titulada «La verruga peruana» le permitió obtener el grado de doctor en medicina y cirugía en Berlín, en mayo de 1877 (Figura 1).

**Figura:** 1. Francisco Puelma Tupper (1850-1933). Museo Nacional de Medicina, Facultad de Medicina, Universidad de Chile.

Fuente: https://n9.cl/1z0rl

Puelma regresó a Chile en 1879, en un momento en que se había iniciado la guerra contra Perú. Participó en los hospitales de sangre, fue nombrado jefe de la Sanidad del Ejército y la Armada, y designado profesor de anatomía patológica de la Escuela de Medicina en 1881. Poco después, incursionó también en la política.

La tesis sobre la verruga peruana fue reproducida en La Revista Médica de Chile en 1878 ^4^^,^^5^. Dicha tesis se basa en las observaciones de diversos autores, a quienes cita con frecuencia. Además, Puelma menciona haber participado en dos autopsias de pacientes que habían estado en Perú y retornaron a Santiago con verrugas.

En los antecedentes históricos, Puelma menciona los escritos sobre verrugas de Tschudi, Hirsh, Smith, Dounon, Renaud y Raimondi, entre otros. Cita al médico peruano Tomás Salazar, quien aprobó y publicó su tesis de doctorado que denominó «Historia de las verrugas» en 1858, aunque lo hace erróneamente, pues lo menciona como M. Salazar y atribuye la publicación al año 1860. Curiosamente, no menciona a su coterráneo, el doctor Nicolás Malo, quien había presentado su tesis sobre la verruga peruana en Santiago, en 1852, como se menciona líneas arriba.

Para la época en que Puelma recolecta el material para su tesis, en 1872, en el Perú existía un intenso debate sobre la naturaleza de la enfermedad de verrugas y su relación con la llamada fiebre anemizante o fiebre de La Oroya. Sin embargo, no hace referencia alguna de las experiencias peruanas. Describe la coexistencia de ambas enfermedades, fiebres y verrugas, pero no se atreve a establecer una conclusión:

*Ademas, aparece la verruga i la fiebre intermitente en la misma comarca i al mismo tiempo, sin que se pueda decir que la primera afección acompañe a la segunda, miéntras que puede decirse que la segunda acompaña a la primera, a lo ménos por ser en la misma comarca. Siempre se encuentra donde la verruga es endémica, la fiebre intermitente; de manera que muchos desgraciados tienen que soportar ambas afecciones. (sic)* (Puelma Tupper, F. VII(11): 275).

Se debe reconocer que la tesis de Puelma es más ordenada que la de Malo, pero refleja una gran confusión propia de la época, cuando aún se iba conociendo la nosografía de las enfermedades y se desconocía la etiopatogenia de la enfermedad de verrugas.

En cuanto a la anatomía patológica, Puelma menciona en su tesis: 

*Solo se han hecho cuatro autopsias de cadáveres de verrugas. Una de ellas fué hecha por el citado Salazar, i para evitar repeticiones la trascribimos aquí. (sic) (Puelma Tupper, F. VII(11): 300).*

Luego de reproducir la autopsia de Salazar, Puelma refiere haber participado en dos autopsias realizadas en Santiago en 1872, cuando aún era estudiante. Los hallazgos mostraron hepatomegalia, esplenomegalia y las lesiones dérmicas características. Sin embargo, también se identificaron otros hallazgos que sugieren que dichos individuos verrucosos no murieron a causa de las verrugas, sino por complicaciones. Actualmente, se sabe que, en la fase de verrugas, el individuo afectado se halla en un estado de inmunodeficiencia por lo que son frecuentes las superinfecciones.

En cuanto a la histología, afirma haber realizado un estudio microscópico de las verrugas y señala lo siguiente:

*...constan de capilares o vasos mas grandes que presentan un tipo cavernoso. Al rededor de estos vasos.se ve un tejido celular que recuerda al embrional, en íntima unión con la pared del vaso i sin división visible do éste. A menudo se ven corpúsculos sanguíneos i cristales de hematina entre las mallas del tejido celular. A veces se hallan pequeños focos hemorrájicos. Todo esto está encerrado en una capa de cutis i de epidermis. La epidermis, empero, está mui engrosada en la parte prominente de la verruga;... (sic)* (Puelma Tupper, F. VII(12): 353).

Puelma no estaba de acuerdo con la denominación de «sarcomas» para las verrugas, tal como lo hicieran Dounon y Renaud, y prefería llamarlas «verrugas sanguíneas», al igual que Tschudi.

Resumiendo, siendo aún estudiante, en 1872, Puelma recopiló información sobre la verruga peruana a partir de la lectura de artículos previos, y participó en las autopsias de dos ciudadanos chilenos que habían retornado a su país con verrugas:

*Nosotros podemos agregar que en Chile no se conoce caso de infección, de verruga a pesar de los muchos paisanos que vuelven del Perú atacados por ella. (sic)* (Puelma Tupper, F. VII(11): 276).

De esta manera, Puelma tuvo el material suficiente para presentarlo como tesis de doctorado, estando en Berlín, en 1877.

Como la tesis de Puelma se publicó en Santiago, en el volumen VII, números 11 y 12 de La Revista Médica de Chile, correspondientes a los años 1878-1879, es probable que esta información no haya llegado al Perú debido a la guerra en curso y al bloqueo del puerto del Callao por parte de la escuadra chilena. En efecto, entre el 5 de abril y el 8 de agosto de 1879 se produjeron incursiones navales por parte de ambos bandos culminando con la pérdida del principal navío peruano en Punta Angamos. Posteriormente, Chile estableció el bloqueo naval del Callao, que se extendió del 10 de abril de 1880 al 17 de enero de 1881, impidiendo la entrada y salida de buques en dicho puerto.

### Luis Sanfurgo Reyes

Nacido en 1859, llegó a Lima con la ocupación de la ciudad por las fuerzas chilenas, como parte del cuerpo de sanidad militar, en la segunda quincena de enero de 1881, y permaneció hasta fines de 1883, cuando se retiraron las tropas invasoras.

Sanfurgo observó que muchos soldados chilenos desarrollaron la verruga peruana, lo que lo motivó a dedicar su tesis a este tema. Esto era previsible, dado que las tropas chilenas incursionaron en los valles verrucógenos.

Su tesis, titulada «La verruga peruana i su tratamiento», presentada como memoria, fue leída en Santiago el 20 de febrero de 1885, lo que le permitió obtener la licenciatura en medicina y farmacia ^6^.

A juzgar por el contenido de su trabajo, Sanfurgo conocía los artículos previamente publicados sobre la verruga peruana, y no escatima en mencionar a sus coterráneos Nicolás Malo y Francisco Puelma Tupper.

La tesis monográfica de Sanfurgo constituye una buena revisión de lo que se conoce sobre verruga peruana hasta entonces, y está redactada de manera ordenada, comparable incluso con el trabajo de Tomás Salazar publicado en 1858, a quien también menciona.

De su memoria se infiere que, en Lima, Sanfurgo prestaba servicios en el Hospital de Santa Sofía, entonces convertido en hospital militar exclusivo para heridos y enfermos del ejército de ocupación chileno ^7^. Este hospital fue construido entre 1872 y 1876, financiado por el empresario francés Auguste Dreyfuss, en memoria de su esposa Sofía Bergmann, fallecida en 1871. Se ubicaba en el local del actual Instituto de Educación Superior Tecnológico Público José Pardo.

Sanfurgo realizó estudios microscópicos y experimentales del agua de la Quebrada de Verrugas, en un intento por hallar un posible agente causal:

*El análisis microscópico no ha dado aun resultados positivos i solo restos de vejetales i algunos infusorios nos han llamado la atencion. Las inyecciones hipodérmicas practicadas con esas aguas en cuyes i conejos no han dado tampoco resultados.* (sic) (Sanfurgo Reyes, L. pág 521).

Sanfurgo tuvo conocimiento de las discusiones sobre la unidad etiológica de la enfermedad, esto es que la fiebre anemizante y la enfermedad de verrugas obedecen al mismo agente, pero no está de acuerdo con esa posición:

*“Algunos han creído encontrar cierta semejanza de desarrollo i de accion entre el ajente de esta enfermedad i el que produce las intermitentes; mas, si esta semejanza puede ser cierta para lo primero, no lo es, en cambio, para lo segundo, pues las lesiones anátomo-patolójicas, los síntomas, el curso i tratamiento i aun la fisonomía misma de ámbas enfermedades son en un todo diferentes; i si por el cambio de lugar o de clima los dos ajentes se destruyen por no poder vivir fuera de las condiciones en que han nacido, no por esto dejan de ser índividualidades patolójicas distintas que modifican el organismo de un modo enteramente diverso.”* (sic) Sanfurgo Reyes, L. pág. 521).

Sanfurgo describe el examen macroscópico al corte de una verruga, pero no realizó estudios microscópicos, lo que se deduce de:

*“Del examen microscópico practicado por los señores Dounon i Renaud, i últimamente por el profesor Izquierdo, resulta que los tumores dérmicos superficiales se orijinan en las papilas mismas i levantan i adelgazan considerablemente la epidérmis que los cubre. Están f ormados por tejido cavernoso de areolas circulares, llenas de sangre durante la vida i vacías despues, i cuyas travículas son muí ricas en células.”* (sic) Sanfurgo Reyes, L. pág. 518).

En este punto, cabe destacar que Sanfurgo se refiere al doctor Vicente Izquierdo, a quien probablemente contactó al finalizar la guerra, en 1884, y de quien obtuvo la información que cita en su tesis. Fue el propio Sanfurgo quien proporciono las muestras de las verrugas para que Izquierdo las examinara, lo que permitió a este último identificar un supuesto «esquizomiceto» como posible agente causal:

*Era, pues, muí posible que la verruga se encontrara tambien en análogas condiciones de jénesis que las intermitentes palúdicas. Influenciados por estas ideas, recojímos con el mayor esmero una cantidad de tumores de las diversas formas, tomados ya durante la vida o ya despues de la muerte de los pacientes í que conservamos en alcohol para confiarlos al profesor don Vicente Izquierdo, quein ha hecho un prolijo estudio de ellos i encontrado, por fin, la causa de esta especial enfermedad en Ia presencia de un esquisomiceto.*

*De la clase de los micrococus, este esquisomiceto afecta muchas veces la forma de un bascillus por agruparse en filas i en número variable, filas que casi nunca son rectilíneas sino en zic-zac o en forma de S. Su tamaño es como la. mitad del de los gonococus i son redondos o lijeramente elípticos cuando están en fila. Se les encuentra en el tejido estructural de los tumores entre las células, formando pequeñas colonias i nunca en el interior de ellas. Encuéntrase tambien en el interior de los capilares i pequeños vasos arteriales i venosos de la piel aparentemente sana i de la que cubre los nudos, en los capilares de éstos i en las del tejido celular subcutáneo, hallándose muchos de esos vasos enteramente trombosados por masas compactas de micrococus que los dilatan irregularmente hasta el punto de asemejarlos a los vasos linfáticos i observándose mui a menudo alrededor de ellos los principios de una proliferacion celular, oríjen de los tumores. Son estos microseres los que, como en la tuberculosis, la lepra, etc., produciendo una irritacion celular en el tejido conjuntivo del cútis i de las mucosas, orijinan los tumores de esta enfermedad i la alteracion de la sangre, pues que con ella circulan. (*sic) (Sanfurgo Reyes, L. pág. 522).

En aquella época, las bacterias eran denominadas «esquizomicetos», del griego: «hongos que se dividen», ya que se pensaba que era un tipo de hongo debido a su reproducción por escisión o división celular. ¿Era microbio alguno lo que vio Izquierdo, según Sanfurgo? ¿Eran bacilos en fila, elípticos y casi elípticos, en filas de zigzag o de S?.

Actualmente se sabe que las bartonelas son bacilos y cocobacilos, pero no se agrupan en filas. Un gonococo mide en promedio 0,8 micras, mientras que la longitud de una *Bartonella* es de 2 a 3 micras. Estas bacterias son intracelulares y se alojan en las células endoteliales del verrucoma, sin formar trombos endovasculares. Por lo tanto, es poco probable que Izquierdo haya observado al verdadero agente causal de la verruga peruana.

Dado que Sanfurgo presentó su tesis el 20 de febrero de 1885, es razonable suponer recibió la información de Izquierdo durante 1884. Curiosamente, aunque Sanfurgo estuvo en Lima entre 1881 y 1883 atendiendo a heridos y enfermos del ejército chileno, e interesado en los casos de verruga entre los soldados, los médicos locales no lo mencionan sino hasta 1898.

Resumiendo, Sanfurgo realizó una descripción detallada de la verruga peruana en su tesis, incorporó la información proporcionada por Izquierdo y dejó entrever una posible causa microbiana de la enfermedad. Sin embargo, la tesis de Sanfurgo pasó desapercibida en el Perú, donde no fue citado sino muchos años después de 1885, año en que ocurrió la autoexperiencia de Carrión.

### Vicente Izquierdo Sanfuentes (1850-1926)

Se formó académicamente en Alemania, donde completó su formación médica y científica. Se graduó de médico en 1879 y retornó a Chile, en medio de la Guerra del Pacífico (1879-1883). Allí revalidó su título de médico y fue nombrado profesor de histología en la Universidad de Chile en 1881. Ese mismo año viajó nuevamente a Alemania y retornó en 1883 (Figura 2).

**Figura 2:** Vicente Izquierdo Sanfuente (1850-1926). Museo Nacional de Medicina, Facultad de Medicina, Universidad de Chile.

Fuente: https://n9.cl/5rzjz

En 1885, Izquierdo publicó un artículo en la prestigiosa revista alemana*Virchows Archiv*, sobre su investigación en la que destacó el hallazgo de un presunto agente causal de la verruga peruana («Spaltpilze bei der “Verruga peruana”»), enfermedad que había despertado su interés por haber afectado a soldados chilenos durante la guerra ^8^. 

En su artículo, Izquierdo menciona cómo obtuvo las muestras:

*Se sabe que la enfermedad infecciosa, endémica en Perú y conocida como “verruga peruana”, no se da en Chile. Los pocos casos que se tratan en los hospitales de Santiago provienen de Perú y en su mayoría son curados. Por lo tanto, es muy difícil obtener material fresco para el examen histológico. Recientemente recibí de Lima algunos trozos de piel de la zona de la rodilla y del prepucio, que mostraban varios nódulos similares a la verruga. El material estaba muy conservado en alcohol y diez trozos fueron tomados después de la muerte de un individuo que había sucumbido a la enfermedad.* (sic, trad.) (Sanfurgo Reyes, L. pág. 522)

Izquierdo destacó, como ya se conocía, la naturaleza vascular de la lesión verrucosa:

*Así pues, los nódulos de la verruga se asemejan en su estructura a los sarcomas, sin que ello sea motivo para considerarlos como tales, como hacen Dounon y Renaud, ya que las verrugas, como veremos en breve, surgen como resultado de la irritación del tejido conjuntivo por un hongo de fisión, que probablemente es peculiar de esta enfermedad. En este sentido son similares a los nódulos de la lepra y de la tuberculosis. Todos los nódulos son muy ricos en vasos sanguíneos; un gran número de capilares recorren el tejido en todas direcciones y una buena parte de ellos están obstruidos por un gran número de bacterias. En algunos nódulos los vasos, especialmente en su parte media, son muy gruesos y están completamente llenos de glóbulos rojos; en otros lugares se desarrolla un verdadero tejido cavernoso, en el que la sangre llena grandes cavidades, separadas entre sí por paredes fibrosas anastomosadas; la similitud con un verdadero angioma cavernoso es completa* (sic, trad.) (Izquierdo V. pág. 413).

Como era de esperar para la época, Izquierdo buscó un agente microbiano en la verruga peruana:

*Es natural que haya buscado hongos de fisión en el producto patológico de una enfermedad infecciosa, utilizando los nuevos métodos que posee la ciencia para detectar la presencia de bacterias en los tejidos. Mis investigaciones me han llevado al descubrimiento de un hongo de fisión de la forma Bacillus, que, por su íntima conexión con el tejido de los nódulos, pensé que era la causa de los productos de la enfermedad, y que probablemente era la causa de la enfermedad misma. Este Bacilo se encuentra tanto en los propios nódulos, como en la piel aparentemente sana que los rodea. Se colorea con diversos colores de anilina, con fucsia, violeta de metilo, azul de metileno, etc., todo no mancha nada o es tan bonito como el violeta de genciana. Ahora aplicamos este color a todos. Las preparaciones de cada uno de los métodos, mediante los cuales se tiñe el bacilo de Koch, se desarrollan completamente en los ácidos, que dejan inalterada la coloración del bacilo tuberculoso. En un trabajo posterior daré más detalles sobre la técnica.*

*Los Bacillus encontrados pueden alcanzar hasta 20 μ de longitud, pero esto es raro; la mayoría miden 8-12 μ, y se pueden encontrar bastante más pequeños. Son algo más gruesos que los bacilos tuberculosos de Koch. La gran diversidad de las longitudes está probablemente relacionada con las diferentes fases de desarrollo o formación de esporas; los más largos se encuentran siempre en el tejido de los nódulos, al igual que los más pequeños, mientras que los de tamaño medio suelen encontrarse en la piel sana o en los vasos sanguíneos cercanos a las úlceras.* (sic, trad.) (Izquierdo V. pág. 414).

Izquierdo describió bacilos y cocos agrupados, extracelulares y trombosando los capilares, exactamente igual a lo que describió Sanfurgo (ver líneas arriba):

*En el tejido de casi todos los nódulos hay siempre una gran cantidad de los hongos de fisión descritos, de todos los tamaños, en grupos entre las células, muy raramente individuales, y nunca los he visto dentro de las células. Estos grupos están formados por más o menos individuos, que a menudo son muy largos y están estrechamente entrelazados. Estas largas formaciones baciliformes casi nunca son rectas, sino que siempre están dobladas en ángulos más o menos agudos, formando un zig-zag o una S. En algunos lugares el tejido está difusamente permeado por cocos individuales o dos o más cocos conectados, como si los microbios se hubieran disuelto completamente en los gránulos que forman. En estos lugares, penetrados difusamente por cocos o pequeñas cadenas de cocos, las células del tejido están destruidas y en su lugar se encuentran densas masas de cocos.*

*En el centro o la periferia de la mayoría de los ganglios examinados encontré un gran número de vasos capilares y probablemente también pequeñas venas que estaban completamente bloqueadas por masas microbianas compactas. Hay verdaderas redes de estos vasos, que parecen estar inyectados con masas de bacterias y, por lo tanto, completamente trombosados.”* (sic, trad.) (Izquierdo V. pág. 415).

De esta descripción se deduce que Sanfurgo recibió esta información de Izquierdo en 1884 y la copió ad literam.

Las conclusiones de Izquierdo fueron las siguientes:

*(1) Los tumores que se forman en la piel de los pacientes que padecen verrugas son verdaderas neoplasias del tejido conjuntivo, que siempre se desarrollan en la piel o en el tejido celular subcutáneo. Nunca son verrugas en sentido anatómico. 2. su estructura es muy similar a la de los sarcomas; en muchos de ellos se encuentran partes formadas únicamente por tejido cavernoso. (3) En estas neoplasias se encuentra en grandes cantidades cierto tipo de hongo de fisión, que o bien se encuentra entre los elementos anatómicos, como células o fibras, o bien está contenido en los vasos sanguíneos, que a veces los obstruyen por completo. (4) El mismo hongo de fisión se encuentra en los vasos sanguíneos (capilares y venas) de la piel sana y del tejido celular subcutáneo. 5. También se encuentra en el tejido y los vasos de la piel que recubre los nódulos. 6) Así pues, en la sangre de los pacientes con verrugas circula un esquizomiceto que, al estimular el tejido conjuntivo, provoca la formación de nuevas células en lugares determinados y específicos, provocando así el desarrollo de los nódulos, que erróneamente se denominan “verrugas”. 7 No se puede suponer que este hongo de fisión sólo se haya desarrollado después de la muerte; el gran número de vasos trombosados por los microbios y su presencia en los vasos de la piel sana indican lo contrario.* (sic, trad.) (Izquierdo V. pág. 418).

Lo descrito por Izquierdo y repetido por Sanfurgo contrasta con lo que se conoció después sobre las bartonelas: son bacterias tipo bacilos o cocobacilos intracelulares, localizadas en los hematíes durante la fase anemizante y en las células endoteliales de las formaciones verrucosas o verrucomas, y no forman agrupaciones que trombosan los vasos sanguíneos.

Era una época en la que se buscaba identificar los agentes causales de las enfermedades conocidas. Así, tenemos que ya se habían realizado los siguientes descubrimientos: cólera del pollo (1868), lepra (1873), amebiasis intestinal (1875), ántrax (1876), gonorrea (1879), fiebre tifoidea (1880), malaria (1880), tuberculosis (1883), cólera (1883), difteria (1884) y tétanos (1885). Entonces, había que intentar descubrir un agente causal de la verruga peruana.

En el Perú, los estudios de anatomía patológica microscópica recién empezaron a desarrollarse sistemáticamente hacia fines del siglo XIX. En 1899, Manuel Tamayo Möller presentó su tesis de bachiller titulada «Histología patológica de la verruga nodular» ^9^. Ese mismo año, Oswaldo Hercelles Monterola presentó su trabajo «Histología patológica del noduloma verrucosos», donde defiende la teoría vascular como origen de la verruga y menciona la existencia de «gérmenes» causantes de la fiebre de La Oroya y las verrugas, aunque aún no se habían identificado ^10^. Poco después, en 1901, Hercelles instaló su nuevo laboratorio de química, hematología, anatomía patológica y bacteriología en el Hospital Dos de Mayo de Lima.

Por otro lado, en los estudios bacteriológicos conducentes a identificar el agente causal de la enfermedad de verrugas, Alberto Barton publicó sus hallazgos de los «cuerpos endoglobulares» en los hematíes de la fase anemizante de la enfermedad de Carrión en 1905 y que fueron confirmados en 1909 ^11^.

Se debe reconocer el esfuerzo de Izquierdo por tratar de hallar un agente etiológico de la verruga peruana en una época en que se descubrían diferentes microorganismos como causantes de enfermedades. Sin embargo, sus hallazgos no fueron confirmados por estudios posteriores. En su contra jugaron tanto las limitaciones técnicas (como la preservación y fijación de las muestras, las tinciones apropiadas, el grado de resolución de los microscopios, etc.), como las limitaciones del conocimiento de la época, en particular la clasificación y tipificación de los microorganismos.

Carrión se inmoló el 5 de octubre de 1885 al inocularse sangre de una lesión de verruga peruana, en su afán por conocer los síntomas prodrómicos de la enfermedad. La evolución de su padecimiento fue registrada detalladamente por sus compañeros, quienes publicaron el relato un año después de su muerte, en octubre de 1886, en el libro que narraba lo ocurrido ^12^.

En la nota biográfica redactada por sus compañeros, se consigna que Carrión, supuestamente, dijo:

*...pues no qué me dá, el ver que individuos como, el médico chileno Izquierdo, que apenas tuvo unos cuantos tumores para ver, se lance á dar opiniones, á escribir sobre ina enfermedad que nadie mejor debía darla a conocer, pues fuera de los trabajos de los DD. Salazar y Velez no he oido hablar de ningún otro nacional;...* (sic) (Medina C. *et al.* pág. 11).

¿Realmente, Carrión supo de las averiguaciones de Izquierdo en 1885? ¿O fue algo que conocieron sus compañeros en los meses siguientes y pusieron dicha frase en labios de Carrión? Es importante definir esto, ya que algunos autores han supuesto que el estudio de Izquierdo fue un acicate para que Carrión procediera a su autoexperiencia. Incluso, hay autores que colocan al nacionalismo por delante para explicar la determinación de Carrión de inocularse.

Sin embargo, esta hipótesis resulta poco convincente. Carrión había reunido nueve historias clínicas de pacientes verrucosos durante los tres años previos, publicó un artículo sobre la orina de los verrucosos, deseaba conocer el pródromo de la enfermedad y se inoculó con el único objetivo de recopilar información para su tesis, ya que se encontraba en el último año de la carrera médica.

El artículo en mención de Izquierdo fue publicado en Berlín, en idioma alemán, en el volumen 99 de la revista correspondiente a marzo de 1885. Carrión se inmoló a comienzos de octubre de ese mismo año. En aquel entonces, la distribución de publicaciones extranjeras se realizaba por vía marítima y la distribución terrestre era lenta. Además, las revistas solo llegaban a los suscriptores, lo que implicaba una inversión que muchos no estaban en condiciones de hacer.

Es muy probable que dicho artículo haya circulado, en el mejor de los casos, de forma oral entre 1885 y 1886. Debe recordarse que, en el Perú, desde mediados del siglo XIX predominaba la influencia de la medicina francesa, mientras que la publicación de Izquierdo fue realizada en una revista alemana. Por tanto, resulta poco probable que Carrión haya llegado a conocer la publicación de Izquierdo.

Es probable que sus compañeros hayan matizado la biografía de Carrión al incluir dicho detalle en la publicación de 1886. Por otro lado, como ya se mencionó, la tesis de Sanfurgo, de 1885, en la que se hace referencia al trabajo de Izquierdo, fue conocida mucho tiempo después, a través de los antecedentes históricos presentados por el doctor Ernesto Odriozola en 1898 ^13^.

En cuanto a la enfermedad de la verruga peruana, hacia fines del siglo XIX y durante las primeras décadas del siglo XX, los estudios nacionales y extranjeros fueron definiendo la nosografía, histopatología, agente bacteriano causal y vector. Hubo varias observaciones y publicaciones en el extranjero realizadas por viajeros y médicos que no fueron conocidas en Perú sino muchos años después. Por ello, no contribuyeron directamente al desarrollo de las investigaciones nacionales sobre la enfermedad de Carrión, con sus fases anemizante y verrucosa.

Las referencias sobre la verruga peruana empezaron a mencionarse después de 1885, año de la autoexperiencia de Carrión. Fue así como empezó a estudiarse las referencias de los cronistas de la Conquista, la verruga de las tropas libertadoras y las experiencias de los viajeros extranjeros. Una de las primeras revisiones fue la del doctor Ernesto Odriozola.

En 1898, el doctor Odriozola publicó su libro «La maladie de Carrion», probablemente el estudio más completo de la época para dicha enfermedad ^13^. En los antecedentes históricos, se refiere a Malo de la siguiente manera:

*Consideramos que esta tesis, a pesar de sus numerosos errores y omisiones, es de gran mérito. No debemos olvidar que es el primer intento sobre el tema y, por imperfecta que sea, demuestra un espíritu de investigación clínica; y las contradicciones que allí se encuentran son sólo las de una experiencia aún rudimentaria de la época.* (sic, trad.) (Medina C. *et al.* pág. 11).

En cuanto a Puelma Tupper, por su tesis de 1877, luego de realizar varias observaciones, concluye lo siguiente:

*No queremos criticar otros errores, que eran muy comprensibles dada la época en que se publicó esta obra. Pero queremos dejar claro que esta tesis parece merecedora de ser tenida en alta estima y el autor demuestra un juicio clínico muy serio.* (sic, trad.) (Odriozola E. pág. 31-32).

Con respecto a Sanfurgo, luego de hacerle objeciones en diversos aspectos de su trabajo, y refiriéndose al tratamiento de las verrugas, Odriozola escribe:

*En el capítulo sobre el tratamiento, afirma, o nos deja menos adivinar, que fue el primero en usar arsénico y yodo, y añade que este último le dio excelentes resultados tanto interna como externamente. Queremos ceñirnos a la cronología de los hechos en este punto y demostrar que hace cuarenta años nuestros antiguos practicantes, Ríos Macedo, Odriozola, Villar, Olaechea, Espinal, etc., usaron arsénico y yodo sin el menor resultado y en cientos de casos.* (sic, trad.) (Odriozola E. pág. 32-33).

Y, sobre la publicación de Izquierdo, Odriozola concluye en:

*Además, el examen bacteriológico no se realizó con el rigor científico requerido. Sus observaciones se centraron en verrugas extirpadas sin precaución y que habían permanecido en alcohol durante mucho tiempo. De esto se desprende claramente que estamos autorizados a formular grandes reservas sobre sus conclusiones. (sic, trad.)* (Odriozola E. pág. 34-35).

Luego de una extensa revisión, histórica, geográfica, epidemiológica y clínica de la enfermedad de verrugas, Odriozola, que era un médico eminentemente clínico, reservó el capítulo de anatomía patológica microscópica para su profesor, el doctor Maurice Letulle, de la Facultad de Medicina de la Universidad de París.

El profesor Letulle, hizo una detallada descripción de la anatomía microscópica y en cuanto al supuesto agente encontrado por Izquierdo fue muy cauto al concluir:

*A la espera de los resultados de los experimentos y cultivos realizados por el profesor Odriozola, podemos preguntarnos, junto con Izquierdo, si el bacilo representado en la lámina VIII, fig. 2, no es el elemento patógeno de la verruga.*

*En el futuro se lo considerará como un bacilo saprofito comparable a los numerosos parásitos de la piel humana, o como el elemento primordial de la enfermedad verrugosa endémica, tan notablemente descrita en el libro que acabamos de leer.* (sic, trad.) (Odriozola E. pág. 201-210).

En conclusión, es importante rescatar el interés de los médicos chilenos Nicolás Malo, Francisco Puelma Tupper, Luis Sanfurgo Reyes y Vicente Izquierdo Sanfuentes por dar a conocer y publicar sobre la enfermedad de verrugas, a pesar de las limitaciones propias de la época (tabla 1). Para ellos era una enfermedad ignota y exótica, y no publicaron sobre el tema después. En consecuencia, sus publicaciones fueron trabajos aisladas que fueron descubiertos posteriormente por los autores nacionales, quienes los mencionaron en la bibliografía de los antecedentes históricos de la enfermedad de verrugas, a medida que las conocieron. Por tanto, ninguna de dichas publicaciones constituyó un aporte sustantivo ni tuvo un impacto directo en las investigaciones médicas peruanas sobre la fiebre anemizante y la fase verrucosa, que hoy se reconocen como dos manifestaciones de una misma enfermedad.

**Tabla 1:** Médicos chilenos que publicaron sobre verruga peruana en el siglo XIX.

	Año de publicación	Tipo de publicación	Lugar	Título	Estuvo en el Perú
Nicolás Malo	1852	Tesis de bachiller	Santiago de Chile	Verruga peruana	Sí
Francisco Puelma Tupper	1879	Tesis doctoral en medicina i farmacia	Berlín	La verruga peruana	No
Luis Sanfurgo Reyes	1885	Memoria para optar el grado de licenciatura en medicina i farmacia	Santiago de Chile	La verruga peruana i su tratamiento	Sí
Vicente Izquierdo Sanfuentes	1885	Artículo	Berlín	Esquizomicetos en la verruga peruana	No
